# A refactoring categorization model for software quality improvement

**DOI:** 10.1371/journal.pone.0293742

**Published:** 2023-11-02

**Authors:** Abdullah Almogahed, Hairulnizam Mahdin, Mazni Omar, Nur Haryani Zakaria, Yeong Hyeon Gu, Mohammed A. Al-masni, Yazid Saif

**Affiliations:** 1 Faculty of Computer Science and Information Technology, Universiti Tun Hussein Onn Malaysia, Parit Raja, Johor, Malaysia; 2 School of Computing, Universiti Utara Malaysia, Sintok, Malaysia; 3 Department of Artificial Intelligence, College of Software & Convergence Technology, Sejong University, Seoul, Republic of Korea; 4 Faculty of Mechanical and Manufacturing Engineering, Universiti Tun Hussein Onn Malaysia, Parit Raja, Johor, Malaysia; West Pomeranian University of Technology, POLAND

## Abstract

Refactoring, a widely adopted technique, has proven effective in facilitating and reducing maintenance activities and costs. Nonetheless, the effects of applying refactoring techniques on software quality exhibit inconsistencies and contradictions, leading to conflicting evidence on their overall benefit. Consequently, software developers face challenges in leveraging these techniques to improve software quality. Moreover, the absence of a categorization model hampers developers’ ability to decide the most suitable refactoring techniques for improving software quality, considering specific design goals. Thus, this study aims to propose a novel refactoring categorization model that categorizes techniques based on their measurable impacts on internal quality attributes. Initially, the most common refactoring techniques used by software practitioners were identified. Subsequently, an experimental study was conducted using five case studies to measure the impacts of refactoring techniques on internal quality attributes. A subsequent multi-case analysis was conducted to analyze these effects across the case studies. The proposed model was developed based on the experimental study results and the subsequent multi-case analysis. The model categorizes refactoring techniques into green, yellow, and red categories. The proposed model, by acting as a guideline, assists developers in understanding the effects of each refactoring technique on quality attributes, allowing them to select appropriate techniques to improve specific quality attributes. Compared to existing studies, the proposed model emerges superior by offering a more granular categorization (green, yellow, and red categories), and its range is wide (including ten refactoring techniques and eleven internal quality attributes). Such granularity not only equips developers with an in-depth understanding of each technique’s impact but also fosters informed decision-making. In addition, the proposed model outperforms current studies and offers a more nuanced understanding, explicitly highlighting areas of strength and concern for each refactoring technique. This enhancement aids developers in better grasping the implications of each refactoring technique on quality attributes. As a result, the model simplifies the decision-making process for developers, saving time and effort that would otherwise be spent weighing the benefits and drawbacks of various refactoring techniques. Furthermore, it has the potential to help reduce maintenance activities and associated costs.

## 1. Introduction

Software refactoring refers to a systematic approach employed to enhance the internal structure of a software system without affecting its functionality [1, 2]. The primary objective of this practice is to improve the quality of software design, leading to reduced maintenance efforts and costs [3]. By improving the structural composition of the software while retaining its intended behavior, software refactoring represents an established solution in this domain [4]. Consequently, it has evolved into a vital component of software development practice, particularly considering the dynamic nature of information technology and user requirements [3].

Presently, the rise of refactoring as a crucial element in software development procedures can be attributed to several aspects, such as evolving needs, adaptability requirements for different situations, and quality inadequacies [5]. As a frequently employed practice, refactoring has become a key method to improve the condition of existing software [6, 7], demonstrating a substantial relationship with the characteristics of software quality [8, 9]. The effectiveness of refactoring techniques in improving quality attributes has been the subject of empirical studies.

However, the relationship between refactoring techniques and software quality attributes is complex [10–12]. Existing literature reveals a multitude of contrasting findings on the impact of refactoring: while some studies affirm positive effects on software quality [13–15], others indicate negative [16, 17] or negligible impacts [18, 19]. The varied outcomes underscore the intricate nature of the correlation between refactoring techniques and software quality [11, 12]. Furthermore, the sequencing of applying refactoring techniques can also yield different effects on quality attributes, complicating the understanding even further [20]. Such inconsistencies present a significant challenge for developers [8, 9]. Picking the right refactoring approach from an array of options, especially in the face of contradictory information, can be overwhelming [21–23]. There’s a clear need for a refactoring categorization model that can guide developers in aligning their design objectives with the desired quality attributes [8].

The current literature lacks models that can assist software engineers in identifying suitable refactoring techniques to improve the targeted design objectives of a software structure [24]. Software engineers need explicit directions on which refactoring approach to employ to realize their targeted design goals [25]. Researchers have emphasized the necessity of exploring diverse facets associated with refactoring, including the development of models that recommend appropriate refactoring techniques [9, 26, 27]. A study [3] highlights the challenge of providing recommendations for specific refactoring techniques as one of the key issues to address in refactoring research. In order to fully leverage the benefits of refactoring techniques and ensure their consistent application, it is necessary to establish a refactoring categorization model that can function as a directive for software engineers in selecting the most appropriate techniques.

Nonetheless, up until the present time, no research efforts have introduced a refactoring categorization model expressly crafted to categorize refactoring techniques and improve the quality characteristics of software systems. Consequently, this research endeavor seeks to address these knowledge gaps by proposing a refactoring categorization model that categorizes refactoring techniques in terms of their impact on internal quality characteristics. This model will equip software engineers with the capability to boost the internal quality aspects of software systems by choosing and applying pertinent refactoring techniques in suitable contexts. By considering their design goals, practitioners can effectively improve specific quality attributes using suitable refactoring techniques. The proposed model functions as a directive, furnishing software engineers with enhanced comprehension of the correlations between refactorings and quality characteristics. It empowers practitioners to make informed decisions in the process of choosing the most suitable refactoring techniques to improve specific quality attributes.

The rest of this paper follows the following structure: Section 2 provides an overview of the existing literature in the field. Section 3 explains the methodology employed in developing the refactoring categorization model. Section 4 presents the results and discussion. The evaluation of the proposed model is presented in Section 5. Section 6 addresses potential validity threats associated with the study. Lastly, in Section 7, the paper concludes by highlighting future research objectives and areas for further investigation.

## 2. Related works

Previous research in this domain has yielded diverse findings, as evidenced by a comprehensive analysis of the relevant literature. The reported results can be categorized into the following groups: (1) several studies have demonstrated a positive impact of refactoring on the quality of software systems [13–15, 28, 29], (2) conversely, some investigations have indicated a negative impact of refactoring techniques on software quality [16, 17], (3) certain studies have claimed that refactoring techniques do not show any noticeable impact on the quality [18, 19], and (4) additionally, the influence of refactoring techniques on software quality is still ambiguous in specific scenarios [30, 31]. These findings demonstrate the varying outcomes observed in the literature, highlighting the complex nature of the correlation between refactoring techniques and the quality of software systems. The utilization of software refactoring as a widely recognized technique has been extensive in improving software quality [21, 32]. However, the effectiveness of software refactoring in enhancing all software quality attributes consistently is not uniformly observed [10–12]. An extensive review of the literature reveals conflicting and inconsistent findings regarding the impact of refactoring on quality attributes. This discrepancy arises due to the diverse effects of different techniques on distinct attributes [11, 12, 26, 33]. Consequently, the empirical support for the advantages of refactoring lacks consensus. The studies demonstrate that varying refactoring techniques can lead to significantly different effects on software quality attributes, sometimes resulting in opposing or conflicting outcomes [11, 12, 21, 22]. Besides, the sequencing of applying refactoring techniques can also yield different effects on quality attributes [20]. Consequently, determining the specific effects of each refactoring technique and their respective influence on quality presents significant obstacles [8, 9]. It is important to emphasize that the failure to identify the specific refactoring techniques applied or the lack of individual application of each technique represents a detrimental practice [11].

The inconsistencies and contradictions surrounding the effects of refactoring on the quality of software systems pose significant challenges for developers seeking to utilize these techniques for quality improvement. A study [34] underscores the complexities that developers encounter when assessing the merits and drawbacks of a range of refactoring techniques, particularly when faced with conflicting information about their efficacy. Moreover, studies [21, 35] stress the daunting task of picking the most fitting refactoring approach from a myriad of possibilities to tackle particular design deficiencies. Additionally, a study [21] points out the challenge of specifying the suitable kinds of refactorings to implement in a distinct scenario. Accordingly, developers can leverage categorization models of refactoring techniques to effectively align their design objectives with the desired impact on software quality attributes [8, 9]. These categorization models enable developers to select the most suitable techniques and apply them strategically in relation to specific quality attributes [8]. This can be realized through a detailed evaluation of the distinct influences each refactoring approach exerts on quality characteristics, factoring in their designated function and implications [12, 36].

Numerous research studies have endeavored to categorize refactoring techniques by assessing their influence on quality characteristics. A study [37] categorized four refactoring techniques (Pull Up Method Superclass, Pull Up Method Subclass, Encapsulate Field, and Extract Method) into five internal quality measurements (LCOM, CBO, NOM, RFC, and NOC) utilizing one small case study. Nonetheless, the scope of techniques for refactoring and attributes of quality considered was limited. A study [38] provided a categorization that served as a guideline for improving code cohesion and coupling. They determined a few refactoring techniques that could enhance these internal quality attributes.

Studies [30, 39, 40] presented categorizations of various refactoring techniques such as Encapsulate Field, Extract Method, Extract Class, and Hide Method based on their quantifiable effects on internal evaluation metrics. However, the categorization performed had limitations regarding the number of refactoring techniques and quality attributes that were considered. Additionally, the categorization was established based on small systems, which may restrict the generalizability of the findings. A study [41] categorized a few refactoring techniques (Encapsulate Field, Extract Class, Hide Method, Extract Method, and Consolidate Conditional Expression) depending on their influences on a few quality characteristics (cohesion, coupling, complexity, and inheritance). They analyzed various C# software system projects to categorize these refactorings. A study [42] examined the effect of ten refactoring techniques individually on internal quality characteristics such as inheritance, complexity, cohesion, and coupling. They categorized these refactoring techniques depending on their impact on the related quality attributes. However, it is important to note that these categorizations varied in terms of the number of techniques and attributes considered, as well as the size and nature of the software systems used in the experiments.

Even though there are a handful of studies that have strived to categorize refactoring techniques based on their effects on certain targeted software quality characteristics [22], these categorizations have been restricted concerning the range of refactoring techniques and quality characteristics included. Due to their incomplete coverage of different techniques and quality attributes, the current categorizations of refactoring techniques show limitations with regard to their comprehensiveness. A number of internal quality characteristics, for example, abstraction, composition, polymorphism, encapsulation, hierarchies, and messaging, were not sufficiently included in these categorizations. Consequently, the debate concerning the influence of refactorings on the quality of software continues to be unsettled, with an abundance of conflicting perspectives still prevailing [12, 36, 43]. Additionally, these studies are missing empirical substantiation from industrial contexts on the choice of refactoring techniques, as their selection appears to be guided by the personal judgment of researchers or through literature survey assessments. These observations highlight the existing research gap in thoroughly assessing the impact of various refactoring techniques on the totality of software quality [8, 11, 12].

In light of these constraints, the primary objective of this research is to establish an expansive categorization model for refactoring techniques. This model intends to categorize frequently implemented refactoring techniques, considering an extensive array of internal quality aspects. The study will carry out a detailed exploration of the dominant refactoring techniques favored by professionals in the existing software development landscape. In addition, it will assess the distinct impacts of the refactoring techniques on internal quality dimensions, encompassing attributes such as encapsulation, composition, abstraction, messaging, polymorphism, and hierarchies.

## 3. Methodology

This part describes the stages of development for the refactoring categorization model. The model is built through five core process stages: (1) identifying the refactoring techniques; (2) identifying quality attributes and metrics; (3) selecting case studies; (4) conducting an experimental study; and (5) conducting a multi-case analysis. The five stages are explained in detail in the sections that follow.

### 3.1. Identifying the refactoring techniques

The primary objective of this study in this section is to identify the most widely used refactoring techniques in software development. A comprehensive refactoring catalog was presented by Fowler et al. [1] that included 68 unique techniques that were thoughtfully organized into six different categories, specifically designed for object-oriented paradigms. To determine the prevalent practices, a thorough examination and dissection of the current body of literature were undertaken. Six systematic literature reviews [9, 11, 44–47] and two systematic mapping studies [12, 48] were examined to identify commonly employed techniques for refactoring in academic research. Additionally, studies [49, 50] reported on frequently used refactoring techniques among software engineering professionals at Microsoft. A study [51] enlisted 13 refactoring techniques that were chosen by former industry developers as the most commonly employed refactorings, representing a broad range of practices. Moreover, a study [52] presented insights on the commonly employed refactoring techniques in industrial scenarios. To capture insights from current industry practices, a recent survey carried out by a study [35] delineated the refactoring techniques most prevalently utilized by industry professionals. By considering these multiple sources of information, this study aims to establish a comprehensive understanding of the refactoring techniques that are most frequently used in both academic and industry contexts.

The examination carried out within this study has culminated in the recognition of the top ten most prevalently utilized refactoring techniques. The following are the ten selected refactoring techniques: (1) Introduce Parameter Object; (2) Extract Method; (3) Extract Interface; (4) Inline Method; (5) Remove Setting Method; (6) Move Method; (7) Move Field; (8) Extract Superclass; (9) Rename Method; and (10) Extract Subclass. These techniques have been determined based on their high frequency of utilization in both academic research and industry practices. They encompass a range of operations aimed at enhancing the structure, organization, and maintainability of software code. By recognizing these commonly employed refactoring techniques, this study contributes to the establishment of best practices and guidelines for software engineers seeking to improve their code quality and design.

### 3.2. Identifying quality attributes and metrics

When considering the selection of appropriate metrics, it is crucial to prioritize those that have undergone empirical validation in earlier research. Metric models, including the Quality Model for Object-Oriented Design (QMOOD), Lorenz and Kidd (L&K), Metrics for Object-Oriented Designs (MOOD), and Chidamber and Kemerer (C&K), were extensively validated through empirical research and widely adopted in object-oriented environments [12, 36, 53–55]. In order to fulfill the aims of this study, it is essential to use a model of quality that can precisely assess the quality of the software and determine how refactoring techniques have an impact on that quality. Therefore, the chosen quality model ought to encompass the ability to gauge diverse internal quality characteristics.

In the context of this study, the QMOOD is chosen as it offers a holistic perspective on assessing the quality of software design [56]. In comparison to metrics that are only concerned with object-oriented design, the QMOOD model, which includes 11 internal quality characteristics, offers a broader perspective on software quality [57]. These attributes proficiently embody the core features of object-oriented systems [56], offering an exhaustive perception of the quality of software design [57]. Additionally, QMOOD metrics are widely used, which simplifies evaluations at the system and class levels and boosts their significance in assessing software designs [55].

As a result, a thorough collection of internal quality attributes and corresponding metrics, derived from the QMOOD model, was chosen for this study. These attributes include abstraction, encapsulation, design size, inheritance, complexity, hierarchies, polymorphism, cohesion, coupling, messaging, and composition. To gauge these attributes, a range of specific metrics were utilized, encompassing the Cohesion Among Methods in a Class (CAM), Design Size in Classes (DSC), Number of Polymorphic Methods (NOP), Number of Methods (NOM), Number of Hierarchies (NOH), Average Number of Ancestors (ANA), Measure of Functional Abstraction (MFA), Direct Class Coupling (DCC), Class Interface Size (CIS), Data Access Metric (DAM), and Measure of Aggregation (MOA). Each of these metrics is tailored to assess a unique aspect of the system: NOM evaluates system complexity; CAM measures the level of cohesion; MFA signifies inheritance levels; DSC is a gauge for design size; DAM estimates the degree of encapsulation; NOH captures hierarchy structures; CIS represents the amount of messaging; ANA assesses levels of abstraction; NOP evaluates the use of polymorphism; MOA reflects the degree of composition; and DCC quantifies the extent of coupling. Table 1 demonstrates how these metrics are calculated to assess internal quality attributes.

**Table 1:** Calculation of the metrics [58].

Internal quality attributes	Metric	Metric calculation
**Design size**	DSC	It counts the total number of classes in a design.
**Hierarchies**	NOH	It counts the number of class hierarchies in a design.
**Abstraction**	ANA	It refers to the average number of classes that a class inherits data from them.
**Encapsulation**	DAM	It can be computed by dividing the number of private attributes by the total number of attributes declared in a class.
**Coupling**	DCC	It is used to count different numbers of classes that a class is directly linked to.
**Cohesion**	CAM	It calculates the relation among methods of a class based on a list of parameters for methods.
**Composition**	MOA	It measures the degree of part-whole relationship that is known through the use of attributes.
**Inheritance**	MFA	It is the ratio between the number of inherited methods by a class and the total number of methods that can be accessed through member methods of the class.
**Polymorphism**	NOP	It is used to compute the total number of methods that can reveal polymorphic behavior.
**Messaging**	CIS	It counts the total number of public methods in a class.
**Complexity**	NOM	It counts the total number of methods in a class.

### 3.3. Selecting case studies

The case studies included in this study were thoughtfully selected from two distinct environments: industrial settings and academic research scenarios. This selection was motivated by the aim of encompassing a wide range of programming skills and expertise, ranging from beginners to seasoned professionals. Furthermore, the case studies were chosen in three different sizes (large, medium, and small). By including case studies from these diverse environments and sizes, the investigations conducted in this study gain comprehensive insights and ensure the results’ applicability to a broader context. For the development of the proposed refactoring categorization model, five specific case studies were included. The selected case studies are detailed in Table 2.

**Table 2:** Detailed information about the case studies.

Case Study	Source	Number of classes	Size	Version	Environment	Programming language
**Bank Management System (BMS)**	[59]	34	Small	1.0	Real-World	Java
**Library Management System (BMS)**	[60]	19	Small	1.0	Academic	Java
**Payroll Management System (PMS)**	[61]	12	Small	1.0	Academic	Java
**jHotDraw**	[62]	250	Medium	5.2	Real-World	Java
**jEdit**	[63]	1153	Large	5.5.0	Real-World	Java

These case studies are summarized below:

- Bank Management System (BMS): The BMS case study revolves around a software system that facilitates various banking operations and processes. It provides insights into the challenges and requirements specific to the banking industry.
- Library Management System (LMS): The LMS case study centers around a software system dedicated to the management and organization of library resources. It represents another real-world scenario.
- Payroll Management System (PMS): This case study focuses on a software system designed for managing payroll-related tasks. It serves as a practical example in a real-world environment.
- jHotDraw: This case study pertains to the jHotDraw framework, which is an open-source graphical editor for creating and manipulating diagrams. It offers insights into the challenges associated with graphical editing and visualization tasks.
- jEdit: The jEdit case study focuses on the jEdit text editor, an open-source software tool primarily used for code editing and programming tasks. It provides valuable insights into the unique requirements and challenges related to text editing and software development.

By including these diverse case studies, this research aims to capture a thorough comprehension of refactoring techniques across different software systems and domains, enabling the development of a robust refactoring categorization model.

### 3.4. Conducting the experimental study

An experimental investigation aims to delve into, characterize, substantiate, and acquire exhaustive knowledge of the procedures, tasks, and features linked to a specific occurrence. The primary objective is to expand existing theoretical frameworks or devise novel strategies to improve present methodologies [64]. In line with this objective, this study carried out a total of 42 distinct experiments aimed at examining the influence of the chosen 10 refactoring techniques on a variety of quality characteristics. These experiments involved performing the refactoring techniques a total of 782 times over five separate case studies. Investigating the unique effects each refactoring technique has on quality attributes was the main objective of these experiments.

The Eclipse platform (available at https://www.eclipse.org/downloads/) was used in this study to run the case studies and conduct the experiments, leveraging its robust framework and ease of integration with other tools. Eclipse is an open-source Integrated Development Environment (IDE) primarily used for Java application development. It offers a wide array of tools and plugins to support various programming languages and platforms, making it a versatile and adaptable tool for software development. Eclipse is renowned for its extensibility, modular architecture, and ability to handle large-scale projects. Its comprehensive coding, debugging, and testing capabilities streamline the development process, assisting developers in efficiently producing high-quality software. On average, case study initialization and setup within the IDE took around 2–3 minutes, while individual runtimes varied based on the complexity of the case studies, ranging from a few seconds for simpler case studies to up to 20 minutes for more intricate ones.

**Fig 1:**
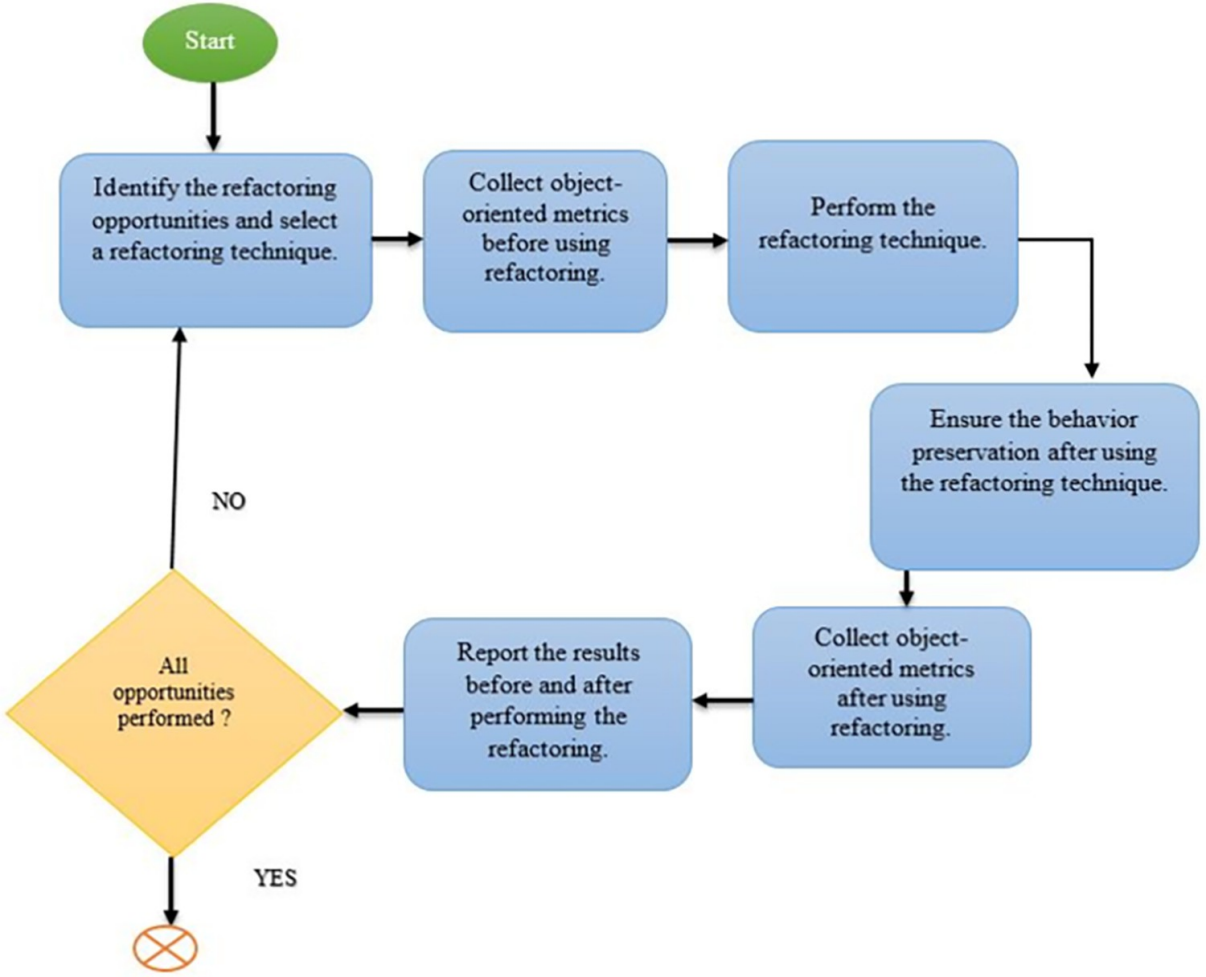
Experimental procedure.

The experiments were designed with careful planning and are outlined in detail in Fig 1, which provides a comprehensive overview of the experimental procedure and methodology to be followed.

By systematically carrying out these experiments, the study aims to gain valuable insights into the impact of every refactoring technique on the quality attributes under investigation. Fowler, in his book [1, 2], identified 23 code smells and 68 refactoring techniques for removing them. He connected each code smell to a specific refactoring technique. In this study, we used the Fowler approach, where each refactoring technique was applied based on the code smell detected in the code. Furthermore, the frequency with which refactoring techniques are used is proportional to the number of code smells detected in the code.

The application of a refactoring technique primarily stems from the identification of code smells, design flaws, or inefficiencies within the software. For instance, the Extract Method refactoring might be chosen when there’s a long method that performs several different operations, making it hard to understand. By extracting portions of this method into their own methods with descriptive names, the code becomes more readable and maintainable. The application of each refactoring technique and the frequency of its application are contingent upon the identification of opportunities, such as code smells or design flaws, that warrant its implementation.

The pertinent internal quality aspects, including inheritance, messaging, design size, encapsulation, complexity, polymorphism, cohesion, hierarchies, abstraction, composition, and coupling, were measured using a collection of metrics. Using the Eclipse Metrics plugin 1.3.8, automatic gathering of the metrics, namely ANA, NOH, DCC, CIS, NOP, NOM, DSC, MFA, CAM, MOA, and DAM, was carried out. Eclipse Metrics 1.3.8, an open-source plugin provided on the Eclipse platform [65], functions as a tool for computing metrics and analyzing dependencies [66, 67]. The vast array of metrics offered by this tool includes those presented in the QMOOD model. Eclipse Metrics 1.3.8 was specifically chosen for this study in order to compile the 11 QMOOD metrics necessary for assessing the internal quality characteristics. It was selected based on its widespread usage in diverse research contexts and its adaptability to major platforms like Mac, Linux, and Windows [68]. By leveraging the capabilities of this widely adopted Java tool, the study aimed to ensure consistency and compatibility in the collection of the necessary metrics for analyzing the quality of the software systems under investigation.

Each refactoring technique has been carried out independently to determine its influence on internal quality characteristics. Specifically, two specific refactoring techniques, namely the Extract Method and Move Method, were performed using the JDeodorant Tool. The JDeodorant Tool, an Eclipse plug-in available at [69], is widely cited and commonly utilized in studies focusing on refactoring [9, 12]. It provides assistance and support for performing refactoring operations, facilitating users’ execution of refactoring transformations [9].

Furthermore, the Rename Method refactoring technique was carried out with the assistance of the Eclipse Refactor plug-in, accessible at https://www.eclipse.org/downloads/. The application of these refactoring techniques utilizing these tools was carefully verified manually to ensure adherence to the mechanics proposed by Fowler. This verification process was essential considering the error susceptibility of current refactoring tools, which could result in the creation of incorrectly refactored code sections [9, 11]. For the other refactoring techniques, they were carried out manually in line with the mechanics suggested by Fowler [1, 2], due to the absence of suitable tools for conducting these specific refactoring operations.

### 3.5. Conducting multi-case analysis

Multi-case analysis is an efficient approach for investigating and understanding complicated phenomena or systems, allowing researchers to acquire insights into theoretical constructs associated with new phenomena [70, 71]. In the present study, the main aim of implementing the multi-case analysis was to categorize the refactoring techniques based on their influence on internal quality aspects. The multi-case analysis entailed gathering and analyzing data from 42 experiments spread throughout five distinct case studies. To execute the multi-case examination, all pertinent measurement data associated with the internal quality characteristics, gathered prior to and following the application of refactoring techniques throughout all of the experiments, was amalgamated into a single pool. The term "pool" is used in this study to refer to the amalgamation of data from all experiments, treated as a unified entity for the purpose of conducting the analysis. The data within the pool were analyzed and compared through cross-case analysis to identify similarities and distinctions among cases, ultimately achieving a sense of generality.

A common practice design has been utilized for categorizing each refactoring technique as per its impact on internal quality characteristics. In this approach, the quality characteristics of the five case studies in the pool were identified, contrasted, and examined in relation to each refactoring technique. The analysis considered how frequently each influence appeared across all experiments. The most frequent effect was then described and chosen to be a part of the refactoring categorization model.

In order to gauge the impact of every individual refactoring on the quality attributes within the pool, a comparative analysis has been conducted between the computed metric values pre- and post-application of the refactoring throughout the case studies. To understand the differences between these calculated values, QMOOD has been used. By subtracting the pre-refactoring metric calculation from its post-refactoring counterpart, the influence attributable to each refactoring technique was determined. A positive discrepancy indicated that the refactoring had a positive effect on the quality attribute; a negative discrepancy indicated a negative effect; and a zero-discrepancy implied that there had been no discernible impact.

Subsequent to this examination, the frequency of each impact (enhancement, degradation, or no impact) on the quality attribute within the pool has been tabulated for every technique of refactoring. The proportion of every impact has been then ascertained. The impact with the highest proportion has been categorized and selected as the emblematic effect for incorporation into the refactoring categorization model. This procedure was repeated for each of the five case studies in the pool for each refactoring technique.

## 4. Results and discussion

The categorization of refactoring techniques in accordance with their impact on internal quality attributes is presented in this section. This categorization approach follows a common practice design methodology and incorporates a multi-case analysis of five case studies, namely jEdit, JHotDraw, PMS, LMS, and BMS. By pooling the results from each case study, we conducted a comprehensive analysis to identify commonalities and differences in the implications of individual refactoring techniques on internal quality characteristics, thus facilitating a general understanding of their impact. The categorization process involves determining the influence of each refactoring technique within the pool of case studies. Specifically, we examine the occurrence rate of each influence and select the effect with the highest occurrence as the representative effect for categorization purposes. This approach allows us to prioritize the effect that is most consistently observable in all five case studies, providing a robust basis for the categorization model. S1 Appendix contains the findings of a multi-case study on the effect of each refactoring technique on each internal quality attribute. Table 3 presents an analytical statistic that illustrates the frequency of application of each refactoring technique across the five case studies. This information serves as an initial exploration of the usage patterns of each technique, providing insights into their relative prevalence within the context of the study.

**Table 3:** Application of refactoring techniques across the five case studies.

No	Refactoring Technique	Case studies					Total
		LMS	BMS	PMS	jHotDraw	jEdit	
**1**	**Extract Method**	0	41	4	51	67	163
**2**	**Inline Method**	2	22	11	56	107	198
**3**	**Move Method**	0	0	1	6	52	59
**4**	**Move Field**	1	0	20	4	22	47
**5**	**Rename Method**	5	11	22	31	40	109
**6**	**Introduce Parameter Object**	1	2	2	8	14	27
**7**	**Remove Setting Method**	0	26	5	35	26	92
**8**	**Extract Subclass**	2	0	1	8	29	40
**9**	**Extract Superclass**	4	4	0	3	10	21
**10**	**Extract Interface**	0	4	2	10	10	26
	**Total**	15	110	68	212	377	782

The frequency of applying a refactoring technique is indicative of the prevalence of a particular code smell or design flaw in the software. For example, if the Extract Method was applied 41 times on BMS software but 0 times on LMS, it suggests that BMS had numerous instances where methods were doing too much and needed decomposition for clarity, whereas LMS did not exhibit this specific issue. The frequency is thus a direct result of the number of occurrences of a specific code issue that the refactoring technique addresses.

This research encompassed 42 separate experiments, throughout which an extensive investigation of refactoring techniques was executed across five different case studies. The 10 selected refactoring techniques were implemented 782 times throughout these explorations. These techniques were then categorized based on their respective impacts on quality characteristics, leading to the identification of three distinct categories:

1. Green Refactoring Techniques (GRT):
  The categorization of these techniques was performed considering their beneficial influence on internal quality attributes. They demonstrated a positive influence on the software system’s quality attributes under consideration.
2. Red Refactoring Techniques (RRT):
  The techniques falling under this category were categorized due to their negative influence on internal quality attributes. These techniques tend to adversely impact the quality attributes of the software system.
3. Yellow Refactoring Techniques (YRT):
  Refactoring techniques in this category were categorized based on the absence of any significant impact on internal quality attributes. These techniques do not bring about noticeable changes in the quality attributes of the software system.

By categorizing the refactoring techniques into these three groups, a structured model was established to better understand their effects on internal quality characteristics. The influence of each refactoring technique (RT) on the quality characteristics is calculated by subtracting the value of the metric on the software system before using RT from the value of the metric after using RT. It was calculated as follows:

### Change in internal quality attribute = metric value post-RT usage—metric value pre-RT usage (1)

**Fig 2:**
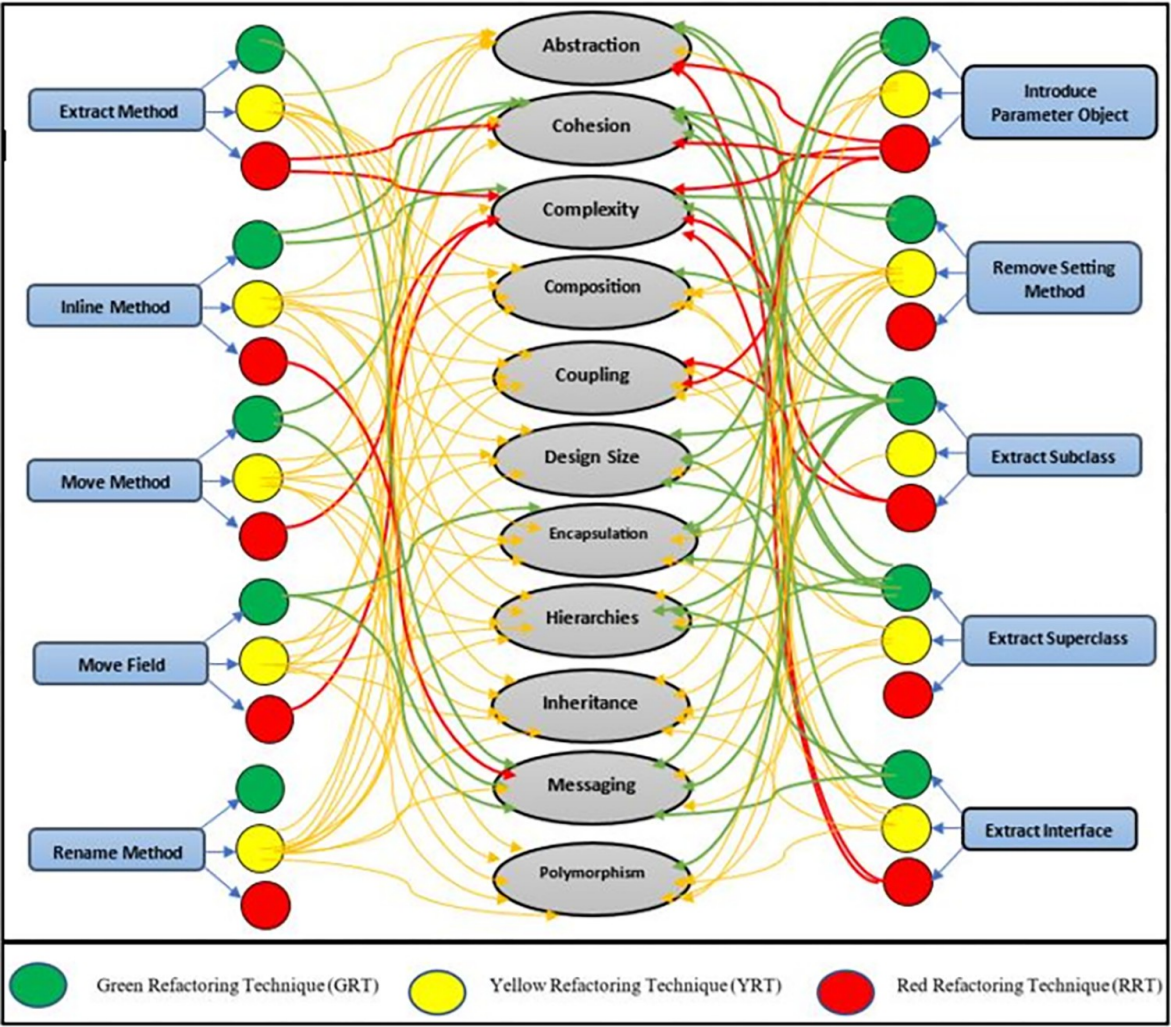
The proposed refactoring categorization model.

In case where the calculated change in the internal quality attribute is a positive number, the refactoring technique improves the quality attribute and is categorized as a green refactoring technique (GRT). If the computed change of the internal quality attribute is negative, the refactoring technique impairs the quality attribute and is categorized as a red refactoring technique (RRT). When the computed change in the internal quality attribute equates to zero, the refactoring technique does not change the quality attribute and is categorized as a yellow refactoring technique (YRT) for this quality attribute. The most frequent impact across the five case studies for each quality attribute served as the basis for categorizing the refactoring techniques. This approach ensured that the refactoring techniques were categorized based on their highest impact on each quality attribute. The representation of the model was achieved by utilizing the strategy of conceptual modeling. Fig 2 shows the proposed refactoring categorization model.

The Extract Method (EM) technique improved messaging and has been categorized as GRT for this attribute. EM was observed to diminish cohesion while augmenting complexity. As a result, for these attributes, it was categorized as RRT. EM had no impact on attributes such as inheritance, abstraction, design size, composition, polymorphism, coupling, hierarchies, or encapsulation. Hence, it was categorized under YRT for these particular attributes.

The Inline Method (IM) technique enhanced cohesion and reduced complexity, leading to its categorization as GRT for these attributes. However, IM showed a decrease in messaging, leading to its categorization as RRT for this attribute. In terms of attributes like abstraction, polymorphism, coupling, inheritance, design size, hierarchies, encapsulation, or composition, IM demonstrated no alterations; hence, it was categorized under YRT for these particular attributes.

The Move Method (MM) technique improved cohesion and messaging, resulting in its categorization as GRT for these attributes. On the other hand, MM led to an escalation in complexity, earning its categorization as RRT for this attribute. In attributes such as abstraction, inheritance, composition, encapsulation, coupling, polymorphism, hierarchies, or design size, MM showed no alterations; therefore, it was categorized as YRT for these particular attributes.

The Move Field (MF) technique augmented encapsulation and messaging; thus, it was placed in the GRT category for these attributes. However, MF amplified complexity, leading to its assignment as RRT for this attribute. For other attributes like design size, composition, hierarchies, abstraction, coupling, polymorphism, cohesion, or inheritance, no modifications were observed after applying MF, which led to its categorization as YRT for these attributes.

The Introduce Parameter Object (IPO) technique augmented design size, encapsulation, and messaging, which led to its inclusion in the GRT category for these attributes. However, IPO lessened abstraction and cohesion while amplifying complexity and coupling, hence its assignment as RRT for these attributes. Regarding attributes like composition, hierarchies, inheritance, or polymorphism, no alterations were noted post-application of IPO, resulting in its categorization as YRT for these attributes.

The Remove Setting Method (RSM) technique bolstered cohesion and curtailed complexity, thus earning its position in the GRT category for these attributes. RSM was found to leave abstraction, messaging, composition, hierarchies, coupling, inheritance, design size, polymorphism, or encapsulation unaltered, which led to its assignment as YRT for these attributes.

The Extract Subclass (ESb) technique positively influenced encapsulation, cohesion, messaging, design size, abstraction, hierarchies, polymorphism, and composition. Hence, it was categorized under the GRT category for these attributes. ESb, however, amplified complexity and coupling, which caused its placement under the RRT category for these attributes. Since ESb had no effect on inheritance, it was categorized as YRT for this attribute.

The Extract Superclass (ESP) technique was observed to decrease complexity while enhancing abstraction, cohesion, design size, encapsulation, and hierarchies. As such, it was assigned to the GRT category for these attributes. ESP left composition, coupling, messaging, inheritance, or polymorphism unchanged, thus leading to its categorization as a YRT for these attributes.

The Extract Interface (EI) technique escalated cohesion, design size, hierarchies, and messaging, thereby being categorized as GRT for these attributes. However, EI was found to diminish abstraction and intensify complexity, which resulted in its categorization as RRT for these aspects. As EI did not cause any alteration in composition, coupling, encapsulation, polymorphism, or inheritance, it has been categorized as YRT for these attributes. Lastly, the Rename Method (RM) technique was categorized as YRT because it had no influence on any quality attribute.

The proposed model categorizes refactoring techniques into three categories: green refactoring techniques (GRT), yellow refactoring techniques (YRT), and red refactoring techniques (RRT). These categories provide software developers with guidelines for selecting suitable refactoring techniques based on their design goals and objectives. The primary goal of refactoring is to enhance software quality. The model categorizes refactoring techniques in accordance with how they impact various internal quality attributes, assisting developers in making informed decisions. It helps them choose appropriate techniques that align with their quality improvement objectives. The model provides a systematic and structured approach for developers to choose the most suitable refactoring techniques based on the specific internal quality attributes they want to improve.

Understanding the relationship between internal quality attributes and the categorization of refactoring techniques is fundamental for the systematic improvement of software. This relationship is integral to guiding developers to choose the right refactoring techniques based on specific quality goals. Internal quality attributes are characteristics of software that, while not directly observable by end-users, play a significant role in maintaining and evolving the software. Examples include messaging, abstraction, encapsulation, cohesion, and coupling. Categorizing refactoring techniques helps group them based on the primary quality attributes they aim to improve or influence. For example, techniques that enhance cohesion were grouped together, while those focusing on messaging were grouped in another category. Each refactoring technique typically targets one or more internal quality attributes. For instance, the Extract Method refactoring technique primarily improves messaging, while the Inline Method technique focuses on enhancing cohesion and reducing complexity.

By categorizing refactoring techniques based on the internal quality attributes they impact, developers gain clarity on which techniques to employ to address specific software quality issues. If a codebase suffers from high complexity, for example, developers can apply the Remove Setting Method, Inline Method, and Extract Superclass refactoring techniques to address the issue. As refactoring techniques are applied based on their categorizations, it’s crucial to evaluate their effects on the targeted internal quality attributes. This evaluation ensures that the techniques are genuinely beneficial and that the categorizations remain accurate. The relationship between internal quality attributes and refactoring technique categorizations can be documented to serve as a valuable knowledge base for development teams. This documentation assists in onboarding new team members and ensures consistency in refactoring practices across the team. In essence, the relationship between internal quality attributes and the categorization of refactoring techniques provides a structured approach to improving software quality. This relationship ensures that refactoring efforts are directed towards tangible and specific quality improvements, maximizing the value of each refactoring action and ensuring the long-term maintainability and evolution of the software.

The categorization of refactoring techniques into GRT, YRT, and RRT provides developers with clear guidance on the potential effects of each technique. This guidance saves developers time and effort that would otherwise be spent on trial and error, ensuring more effective decision-making. It helps them understand the trade-offs involved in applying a specific refactoring. By considering the implications for attributes like messaging, cohesion, complexity, and more, developers can make better decisions during the refactoring process. Different software systems have unique design goals and requirements. The proposed model allows developers to select refactoring techniques based on the specific attributes they want to improve or maintain. The proposed model helps them identify techniques that have positive or neutral effects on desired attributes while being aware of any potential negative impacts.

Refactoring is a resource-intensive process, and developers need to allocate their time and effort wisely. The model’s categorization assists in resource allocation by highlighting techniques that are likely to have a significant positive impact (GRT), techniques that have no significant impact (YRT), and techniques that might have trade-offs (RRT). This empowers developers to focus their efforts on the most beneficial techniques for their particular software system. The results of the model provide insights into how each refactoring technique affects different internal quality attributes. This knowledge improves developers’ understanding of software design principles and the consequences of applying specific refactorings. Furthermore, the model can serve as a documentation resource for future reference, enabling developers to make informed decisions during maintenance and evolution phases.

Software systems evolve over time, and refactoring plays a crucial role in managing complexity and maintaining code quality. The proposed model facilitates codebase evolution by providing developers with a categorization that helps them identify the most appropriate techniques to apply. This supports long-term maintainability and enables developers to adapt the codebase to changing requirements efficiently. The proposed model encourages consistent practices across development teams. This improves collaboration, as team members can better understand each other’s code due to consistent refactoring patterns. With a well-defined categorization model, developers can quickly identify the most appropriate refactoring techniques for a given situation. This streamlines the refactoring process and allows them to focus more on adding value to the software than dealing with code issues. The model can be used to document the rationale behind choosing specific refactoring techniques, creating a knowledge base for future reference and knowledge sharing.

Modern IDEs and other software engineering tools can benefit from the categorization. For instance, an IDE might provide refactoring suggestions based on the detected internal quality attributes that need improvement in the codebase. By understanding which internal quality attributes a specific refactoring technique targets, developers can predict the potential impacts of applying that technique, leading to more informed decisions.

In summary, the proposed model for refactoring categorization offers several important benefits. It enhances software quality by guiding developers in selecting suitable techniques for improvement. The categorization provides clear guidance, enables customization, optimizes resource allocation, improves design understanding, and supports codebase evolution and maintenance. Software developers can significantly raise the quality of their systems by utilizing this model, which helps them make more informed decisions during the refactoring process.

## 5. Evaluation of the proposed refactoring categorization model

In this section, we critically evaluate the proposed model in this study by juxtaposing it with the previous studies in the field of software refactoring, as shown in Table 4. The objective is to assess the model’s depth, breadth, and contribution to advancing our understanding of software quality enhancement through refactoring techniques. In addition, evaluating the proposed model against previous studies requires considering several factors, such as the model’s comprehensiveness, specificity, applicability, and clarity.

**Table 4:** A comparative analysis of the proposed model and previous relevant studies.

Study	Refactoring techniques	Quality attributes
**Bois & Mens [37]**	1) Extract Method 2) Encapsulate Field 3) Pull Up Method	1) NOM 2) NOC 3) CBO 4) RFC 5) LCOM
**Bois et al. [38]**	1) Extract Method 2) Move Method 3) Replace Method with Method Object 4)Replace Data Value with Object Extract Class	1) Cohesion 2) Coupling
**Elish & Alshayeb [30]**	1) Encapsulate Field 2) Extract Method 3) Consolidate Conditional Expression 4) Hide Method 5) Extract Class	1) RFC 2) FOUT 3) WMC 4) NOM 5) LOC
**Elish & Alshayeb [39]**	1) Encapsulate Field 2) Extract Method 3) Hide Method 4) Inline Method 5) Remove Setting Method 6) Extract Class	1) Inheritance 2) Coupling 3) Size/complexity 4) Cohesion
**Elish & Alshayeb [40]**	1) Compose Method 2) Form Template Method 3) Unify Interfaces 4) Chain Constructors 5) Introduce Null Object	1) Inheritance 2) Coupling 3) Size/complexity 4) Cohesion
**Malhotra & Chug [41]**	1) Consolidate Conditional Expression 2) Encapsulate Field 3) Extract Method 4) Extract Class 5) Hide Method	Inheritance Complexity Coupling Cohesion
**Malhotra & Jain [72]**	1) Replace Constructor with Factory 2) Replace Constructor with Builder 3) Wrap return value 4) Encapsulate Field	1) Complexity 2) Coupling 3) Cohesion 4) Inheritance
**The propsed model in this study**	1) Extract Method 2) Inline Method 3) Move Method 4) Move Field 5) Rename Method 6) Introduce Parameter Object 7) Remove Setting Method 8) Extract Subclass 9) Extract Superclass 10) Extract Interface	1) Abstraction 2) Encapsulation 3) Design Size 4) Inheritance 5) Complexity 6) Hierarchies 7) Polymorphism 8) Cohesion 9) Coupling 10) Messaging 11) Composition

In order to conduct a comprehensive analysis, it is imperative to systematically assess the proposed model in comparison to the aforementioned studies, as presented in Table 4.

### 5.1. Scope of refactoring techniques

One significant dimension of evaluation pertains to the scope of refactoring techniques encompassed by the proposed model in comparison to prior research endeavors. A meticulous examination reveals that the proposed model offers a comprehensive inventory of refactoring techniques, including both traditional strategies and more recent developments. Notably, the model extends beyond the scope of singular studies such as [37] or [40], which emphasize specific subsets of techniques. This expanded repertoire suggests that the proposed model possesses versatility and adaptability to accommodate a broader array of software development scenarios.

### 5.2. Depth of quality attributes

Another critical aspect of the evaluation is the depth and diversity of quality attributes considered by the proposed model in comparison to the previous studies. Our analysis reveals that the model adopts a multi-dimensional approach by encompassing a comprehensive set of internal quality attributes. Attributes such as abstraction, encapsulation, design size, hierarchies, polymorphism, composition, and messaging are introduced, illustrating the model’s commitment to a nuanced appraisal of software quality. This depth surpasses the evaluation metrics typically explored in previous studies, which often revolve around cohesion, coupling, and complexity. Thus, the proposed model emerges as a more holistic model for quality assessment.

### 5.3. Specificity

The proposed model seems to be general in some areas, targeting a broader application. Some previous studies, like [40], are more specific in their chosen refactoring techniques. While specificity can be valuable in certain contexts, a more generalized approach like that of the proposed model may offer wider applicability.

### 5.4. Applicability

Due to its broad scope, the proposed model can be more applicable to a wide range of software projects and contexts. Some previous studies might be more suited for specific scenarios or challenges in software development due to their focused approach. For instance, the techniques in [41] are very targeted and might be more applicable in certain software contexts than the broader techniques in the proposed model.

### 5.5. Clarity and structure

By categorizing techniques based on their impact on quality attributes, the proposed model provides a clear structure for decision-making. Previous studies tend to list techniques and associated quality attributes but may not provide as structured a guideline for applying them as the proposed model.

### 5.6. Relevance to modern software development

The proposed model introduces techniques that seem relevant to contemporary software development challenges. Earlier studies, given the time they were written, might not encompass the nuances and challenges of modern software development fully. The proposed model, being more recent, could potentially bridge this gap.

### 5.7. Implications and contributions

In light of the foregoing evaluation, several implications arise regarding the proposed model’s contributions to the domain of software engineering research. Firstly, it underscores the model’s potential to amalgamate and build upon the foundations laid by previous studies. By offering a more expansive spectrum of refactoring techniques and quality attributes, the model seeks to mitigate the disparities and ambiguities that have characterized prior research findings. The proposed model represents a notable advancement in the discourse of software refactoring. Its synthesis of insights from previous research, coupled with its inclusive approach, posits it as a unified model for refining software quality. However, its ultimate impact hinges on empirical validation and its capacity to yield consistent improvements in software design and maintenance practices.

## 6. Threats to validity

This section addresses the potential threats to the validity of the study and discusses how they were mitigated to enhance confidence in the research outcomes. The threats to validity are categorized according to the framework proposed by studies [73, 74], encompassing construct validity, conclusion validity, internal validity, and external validity. Each of these threats and their corresponding mitigation strategies are presented below.

### 6.1. Construct validity

Threats to construct validity focus on the harmony between the theoretical construct and the observational measures applied, particularly concerning the measurement methods used. In the current research, we opted for the 10 most frequently used refactoring techniques, drawing upon an in-depth literature survey and an exploratory study with active software professionals. This method was intended to avoid excessive subjectivity and bias during the selection process. Furthermore, the application of selected refactoring techniques strictly followed Fowler’s recommendations, thereby ensuring consistency with accepted practices. For assessing the influence of the refactoring techniques on internal quality attributes, we deployed a recognized and credible quality model, namely QMOOD.

### 6.2. Conclusion validity

Conclusion validity threats pertain to the correlation between the applied intervention and the observed results. They gauge the replicability of the research findings when other investigators undertake a similar study using the identical protocol. To mitigate these threats, this research conducted an extensive series of 42 independent experiments spread over five distinctive case studies (jEdit, JHotDraw, BMS, LMS, and PMS) during the phase of model construction. This wide-ranging experimentation endows sufficient empirical support to formulate reliable deductions. Moreover, the thorough delineation of the experimental process bolsters future attempts to recreate this study.

### 6.3. Internal validity

The accuracy of the established, related connection between an approach and its outcomes is known as internal validity. The case studies incorporated in this investigation are largely indicative of standard software refactoring contexts, hence boosting the internal validity of this research. Each case study was subjected to refactoring treatment exclusively to segregate the impact of the employed techniques. All of the case studies maintained the same treatment settings. Detailed documentation of the post-refactoring state of each case study was maintained to aid in computing the object-oriented metrics. The experimental execution began with smaller-scale case studies, gradually advancing to those of larger scale, allowing for an iterative learning and consistency assurance process for the researcher.

### 6.4. External validity

External validity is concerned with the applicability or transferability of the study’s results. In this research, steps were taken to bolster external validity by performing experiments on an assortment of case studies drawn from academic and real-world sources, consisting of Java software systems of varying domains and scales. Despite the emphasis on Java projects due to its significant standing both in industrial and academic contexts, it is acknowledged that, given the variety of refactoring techniques and tool support, the results may not extrapolate correctly to other programming languages. As a result, it is encouraged that future research explore these topics in other programming languages, such as JavaScript and Python, to increase the breadth of the results’ applicability. By addressing the aforementioned potential validity threats via a rigorous methodology and meticulous consideration of various aspects, this research seeks to offer robust and dependable insights concerning the impacts of refactoring techniques on internal quality attributes.

## 7. Conclusions and future directions

In the software industry, maintaining high software quality is of paramount importance, as low-quality software can lead to increased complexity and present significant maintenance challenges. As a fundamental practice in software maintenance and evolution, software refactoring is widely acknowledged for its ability to enhance the quality of software systems. However, the impact of applying refactoring techniques to different software quality attributes can vary, which poses challenges for developers seeking to improve software quality through refactoring.

To overcome these challenges, this study introduces a refactoring categorization model with the goal of enhancing internal quality attributes. The model identifies the prevalent refactoring techniques and quality attributes. Furthermore, the study incorporates five diverse case studies of varying sizes to conduct comprehensive experiments, examining the specific impacts of the 10 selected refactoring techniques on the quality attributes. Across the course of these five case studies, refactoring techniques were applied a total of 782 times.

The proposed refactoring categorization model was developed using the insights gained from the experimental study in conjunction with a multi-case analysis. The model categorizes the 10 most frequently used refactoring techniques into three categories (green, yellow, and red), reflecting their impacts on 11 quality attributes. Serving as a practical guideline, the model assists software developers in selecting suitable refactoring techniques to enhance software quality. By offering empirical evidence and insights, the model lightens the load for developers who otherwise would need to evaluate the trade-offs and conflicts among different refactoring techniques. Ultimately, the model aids in mitigating risks associated with software maintenance costs and effort.

In future work, the proposed refactoring categorization model will be expanded to encompass a broader set of commonly used refactoring techniques. To assess its effectiveness and suitability, the model will undergo expert reviews involving both academic and industrial experts. This evaluation will provide valuable feedback and improvements to refine the model further. Additionally, the model will undergo empirical evaluation through diverse case studies conducted by domain experts. This real-world testing will serve to validate the model’s applicability and demonstrate its benefits in practical software development scenarios. The insights gained from this extensive evaluation process will enhance the model’s robustness and its potential to assist software developers in making informed decisions for improving software quality. Furthermore, future studies can look into integrating the refactoring categorization model with real-time software monitoring tools. This would allow developers to receive real-time recommendations on refactoring techniques as they code. Based on the categorization models, there’s potential to develop tools or plugins for popular IDEs that can assist developers in choosing refactoring techniques aligned with desired quality outcomes.

## Supporting information

S1 Appendix The multi-case analysis results are summarized, which show how each refactoring technique affected the different internal quality attributes. (DOCX)
