# Supplementary material for: A refactoring categorization model for software quality improvement
Source: PLoS One. 2023 Nov 2;18(11):e0293742. doi: 10.1371/journal.pone.0293742 (PMC10621946; doi:10.1371/journal.pone.0293742)
Supplement: S1 Appendix — (DOCX) [file pone.0293742.s001.docx]

**Appendix A.**

The multi-case analysis results are summarized in the following tables, which show how each refactoring technique affected the different internal quality attributes. With the exception of the complexity and coupling attributes, an increase in the internal quality attribute is denoted by an upward arrow (↑). Contrarily, the downward arrow (↓) denotes a decline or deterioration in the internal quality attribute (with the exception of complexity and coupling). The dash symbol (−) signifies that the quality attribute has not changed.

**Table A1: Multi-case analysis of the Extract Method**

| **Internal Quality Attribute** | **Case Study** | **PMS** | **LMS** | **BMS** | **jHotDraw** | **jEdit** | **Total** | **Effect%** |
| --- | --- | --- | --- | --- | --- | --- | --- | --- |
|  | Times | 4 | 0 | 41 | 51 | 67 | 163 |  |
|  | Impact |  |  |  |  |  |  |  |
| **Design Size (DSC)** | 🡩 | 0 | 0 | 0 | 0 | 0 | 0 | 0.00% |
|  | − | 4 | 0 | 41 | 51 | 67 | 163 | 100.00% |
|  | 🡫 | 0 | 0 | 0 | 0 | 0 | 0 | 0.00% |
| **Inheritance (MFA)** | 🡩 | 0 | 0 | 0 | 0 | 0 | 0 | 0.00% |
|  | − | 4 | 0 | 41 | 51 | 67 | 163 | 100.00% |
|  | 🡫 | 0 | 0 | 0 | 0 | 0 | 0 | 0.00% |
| **Messaging (CIS)** | 🡩 | 4 | 0 | 41 | 17 | 20 | 82 | 50.30% |
|  | − | 0 | 0 | 0 | 34 | 47 | 81 | 49.70% |
|  | 🡫 | 0 | 0 | 0 | 0 | 0 | 0 | 0.00% |
| **Composition (MOA)** | 🡩 | 0 | 0 | 0 | 0 | 0 | 0 | 0.00% |
|  | − | 4 | 0 | 41 | 51 | 67 | 163 | 100.00% |
|  | 🡫 | 0 | 0 | 0 | 0 | 0 | 0 | 0.00% |
| **Encapsulation (DAM)** | 🡩 | 0 | 0 | 0 | 0 | 0 | 0 | 0.00% |
|  | − | 4 | 0 | 41 | 51 | 67 | 163 | 100.00% |
|  | 🡫 | 0 | 0 | 0 | 0 | 0 | 0 | 0.00% |
| **Polymorphism (NOP)** | 🡩 | 0 | 0 | 0 | 0 | 0 | 0 | 0.00% |
|  | − | 4 | 0 | 41 | 51 | 67 | 163 | 100.00% |
|  | 🡫 | 0 | 0 | 0 | 0 | 0 | 0 | 0.00% |
| **Cohesion (CAM)** | 🡩 | 0 | 0 | 0 | 20 | 19 | 39 | 23.93% |
|  | − | 0 | 0 | 4 | 11 | 23 | 38 | 23.31% |
|  | 🡫 | 4 | 0 | 37 | 20 | 25 | 86 | 52.76% |
| **Coupling (DCC)** | 🡩 | 0 | 0 | 0 | 2 | 5 | 7 | 4.30% |
|  | − | 4 | 0 | 41 | 49 | 62 | 156 | 95.70% |
|  | 🡫 | 0 | 0 | 0 | 0 | 0 | 0 | 0.00% |
| **Hierarchies (NOH)** | 🡩 | 0 | 0 | 0 | 0 | 0 | 0 | 0.00% |
|  | − | 4 | 0 | 41 | 51 | 67 | 163 | 100.00% |
|  | 🡫 | 0 | 0 | 0 | 0 | 0 | 0 | 0.00% |
| **Abstraction (ANA)** | 🡩 | 0 | 0 | 0 | 0 | 0 | 0 | 0.00% |
|  | − | 4 | 0 | 41 | 51 | 67 | 163 | 100.00% |
|  | 🡫 | 0 | 0 | 0 | 0 | 0 | 0 | 0.00% |
| **Complexity (NOM)** | 🡩 | 4 | 0 | 41 | 45 | 48 | 138 | 84.66% |
|  | − | 0 | 0 | 0 | 6 | 19 | 25 | 15.34% |
|  | 🡫 | 0 | 0 | 0 | 0 | 0 | 0 | 0.00% |

**Table A2: Multi-case analysis of the Inline Method**

| **Internal Quality Attribute** | **Case Study** | **PMS** | **LMS** | **BMS** | **jHotDraw** | **jEdit** | **Total** | **Effect%** |
| --- | --- | --- | --- | --- | --- | --- | --- | --- |
|  | Times | 11 | 2 | 22 | 56 | 107 | 198 |  |
|  | Impact |  |  |  |  |  |  |  |
| **Design Size (DSC)** | 🡩 | 0 | 0 | 0 | 0 | 0 | 0 | 0.00% |
|  | − | 11 | 2 | 22 | 56 | 107 | 198 | 100.00% |
|  | 🡫 | 0 | 0 | 0 | 0 | 0 | 0 | 0.00% |
| **Inheritance (MFA)** | 🡩 | 0 | 0 | 0 | 0 | 0 | 0 | 0.00% |
|  | − | 11 | 2 | 22 | 56 | 107 | 198 | 100.00% |
|  | 🡫 | 0 | 0 | 0 | 0 | 0 | 0 | 0.00% |
| **Messaging (CIS)** | 🡩 | 0 | 0 | 0 | 0 | 0 | 0 | 0.00% |
|  | − | 0 | 0 | 0 | 21 | 5 | 25 | 13.06% |
|  | 🡫 | 11 | 2 | 22 | 35 | 102 | 173 | 86.94% |
| **Composition (MOA)** | 🡩 | 0 | 0 | 0 | 0 | 0 | 0 | 0.00% |
|  | − | 11 | 2 | 22 | 56 | 107 | 198 | 100.00% |
|  | 🡫 | 0 | 0 | 0 | 0 | 0 | 0 | 0.00% |
| **Encapsulation (DAM)** | 🡩 | 0 | 0 | 0 | 0 | 0 | 0 | 0.00% |
|  | − | 10 | 2 | 4 | 44 | 53 | 113 | 56.78% |
|  | 🡫 | 1 | 0 | 18 | 12 | 54 | 85 | 43.21% |
| **Polymorphism (NOP)** | 🡩 | 0 | 0 | 0 | 0 | 0 | 0 | 0.00% |
|  | − | 11 | 2 | 22 | 56 | 107 | 198 | 100.00% |
|  | 🡫 | 0 | 0 | 0 | 0 | 0 | 0 | 0.00% |
| **Cohesion (CAM)** | 🡩 | 10 | 0 | 22 | 50 | 80 | 162 | 81.40% |
|  | − | 1 | 2 | 0 | 6 | 27 | 36 | 18.60% |
|  | 🡫 | 0 | 0 | 0 | 0 | 0 | 0 | 0.00% |
| **Coupling (DCC)** | 🡩 | 0 | 0 | 0 | 0 | 0 | 0 | 0.00% |
|  | − | 11 | 2 | 22 | 56 | 107 | 198 | 100.00% |
|  | 🡫 | 0 | 0 | 0 | 0 | 0 | 0 | 0.00% |
| **Hierarchies (NOH)** | 🡩 | 0 | 0 | 0 | 0 | 0 | 0 | 0.00% |
|  | − | 11 | 2 | 22 | 56 | 107 | 198 | 100.00% |
|  | 🡫 | 0 | 0 | 0 | 0 | 0 | 0 | 0.00% |
| **Abstraction (ANA)** | 🡩 | 0 | 0 | 0 | 0 | 0 | 0 | 0.00% |
|  | − | 11 | 2 | 22 | 56 | 107 | 198 | 100.00% |
|  | 🡫 | 0 | 0 | 0 | 0 | 0 | 0 | 0.00% |
| **Complexity (NOM)** | 🡩 | 0 | 0 | 0 | 0 | 0 | 0 | 0.00% |
|  | − | 1 | 2 | 0 | 0 | 14 | 17 | 8.54% |
|  | 🡫 | 10 | 0 | 22 | 56 | 93 | 181 | 91.46% |

**Table A3: Multi-case analysis of the Move Method**

| **Internal Quality Attribute** | **Case Study** | **PMS** | **LMS** | **BMS** | **jHotDraw** | **jEdit** | **Total** | **Effect%** |
| --- | --- | --- | --- | --- | --- | --- | --- | --- |
|  | Times | 1 | 0 | 0 | 6 | 52 | 59 |  |
|  | Impact |  |  |  |  |  |  |  |
| **Design Size (DSC)** | 🡩 | 0 | 0 | 0 | 0 | 0 | 0 | 0.00% |
|  | − | 1 | 0 | 0 | 6 | 52 | 59 | 100.00% |
|  | 🡫 | 0 | 0 | 0 | 0 | 0 | 0 | 0.00% |
| **Inheritance (MFA)** | 🡩 | 0 | 0 | 0 | 0 | 0 | 0 | 0.00% |
|  | − | 1 | 0 | 0 | 6 | 52 | 59 | 100.00% |
|  | 🡫 | 0 | 0 | 0 | 0 | 0 | 0 | 0.00% |
| **Messaging (CIS)** | 🡩 | 1 | 0 | 0 | 4 | 36 | 41 | 69.50% |
|  | − | 0 | 0 | 0 | 2 | 16 | 18 | 30.50% |
|  | 🡫 | 0 | 0 | 0 | 0 | 0 | 0 | 0.00% |
| **Composition (MOA)** | 🡩 | 0 | 0 | 0 | 0 | 0 | 0 | 0.00% |
|  | − | 1 | 0 | 0 | 6 | 52 | 59 | 100.00% |
|  | 🡫 | 0 | 0 | 0 | 0 | 0 | 0 | 0.00% |
| **Encapsulation (DAM)** | 🡩 | 0 | 0 | 0 | 0 | 0 | 0 | 0.00% |
|  | − | 1 | 0 | 0 | 6 | 52 | 59 | 100.00% |
|  | 🡫 | 0 | 0 | 0 | 0 | 0 | 0 | 0.00% |
| **Polymorphism (NOP)** | 🡩 | 0 | 0 | 0 | 0 | 0 | 0 | 0.00% |
|  | − | 1 | 0 | 0 | 6 | 52 | 59 | 100.00% |
|  | 🡫 | 0 | 0 | 0 | 0 | 0 | 0 | 0.00% |
| **Cohesion (CAM)** | 🡩 | 1 | 0 | 0 | 1 | 32 | 34 | 57.62% |
|  | − | 0 | 0 | 0 | 0 | 0 | 0 | 0.00% |
|  | 🡫 | 0 | 0 | 0 | 5 | 20 | 25 | 42.38% |
| **Coupling (DCC)** | 🡩 | 0 | 0 | 0 | 1 | 11 | 12 | 20.33% |
|  | − | 1 | 0 | 0 | 5 | 41 | 47 | 79.67% |
|  | 🡫 | 0 | 0 | 0 | 0 | 0 | 0 | 0.00% |
| **Hierarchies (NOH)** | 🡩 | 0 | 0 | 0 | 0 | 0 | 0 | 0.00% |
|  | − | 1 | 0 | 0 | 6 | 52 | 59 | 100.00% |
|  | 🡫 | 0 | 0 | 0 | 0 | 0 | 0 | 0.00% |
| **Abstraction (ANA)** | 🡩 | 0 | 0 | 0 | 0 | 0 | 0 | 0.00% |
|  | − | 1 | 0 | 0 | 6 | 52 | 59 | 100.00% |
|  | 🡫 | 0 | 0 | 0 | 0 | 0 | 0 | 0.00% |
| **Complexity (NOM)** | 🡩 | 1 | 0 | 0 | 2 | 37 | 40 | 67.80% |
|  | − | 0 | 0 | 0 | 4 | 15 | 19 | 32.20% |
|  | 🡫 | 0 | 0 | 0 | 0 | 0 | 0 | 0.00% |

**Table A4: Multi-case analysis of the Move Field**

| **Internal Quality Attribute** | **Case Study** | **PMS** | **LMS** | **BMS** | **jHotDraw** | **jEdit** | **Total** | **Effect%** |
| --- | --- | --- | --- | --- | --- | --- | --- | --- |
|  | Times | 20 | 1 | 0 | 4 | 22 | 47 |  |
|  | Impact |  |  |  |  |  |  |  |
| **Design Size (DSC)** | 🡩 | 0 | 0 | 0 | 0 | 0 | 0 | 0.00% |
|  | − | 20 | 1 | 0 | 4 | 22 | 47 | 100.00% |
|  | 🡫 | 0 | 0 | 0 | 0 | 0 | 0 | 0.00% |
| **Inheritance (MFA)** | 🡩 | 0 | 0 | 0 | 0 | 0 | 0 | 0.00% |
|  | − | 20 | 1 | 0 | 4 | 22 | 47 | 100.00% |
|  | 🡫 | 0 | 0 | 0 | 0 | 0 | 0 | 0.00% |
| **Messaging (CIS)** | 🡩 | 0 | 0 | 0 | 4 | 22 | 26 | 55.32% |
|  | − | 20 | 1 | 0 | 0 | 0 | 21 | 44.68% |
|  | 🡫 | 0 | 0 | 0 | 0 | 0 | 0 | 0.00% |
| **Composition (MOA)** | 🡩 | 0 | 0 | 0 | 0 | 0 | 0 | 0.00% |
|  | − | 20 | 1 | 0 | 4 | 22 | 47 | 100.00% |
|  | 🡫 | 0 | 0 | 0 | 0 | 0 | 0 | 0.00% |
| **Encapsulation (DAM)** | 🡩 | 17 | 0 | 0 | 0 | 9 | 26 | 55.33% |
|  | − | 0 | 0 | 0 | 4 | 0 | 4 | 8.50% |
|  | 🡫 | 3 | 1 | 0 | 0 | 13 | 17 | 36.17% |
| **Polymorphism (NOP)** | 🡩 | 0 | 0 | 0 | 0 | 0 | 0 | 0.00% |
|  | − | 20 | 1 | 0 | 4 | 22 | 47 | 100.00% |
|  | 🡫 | 0 | 0 | 0 | 0 | 0 | 0 | 0.00% |
| **Cohesion (CAM)** | 🡩 | 0 | 0 | 0 | 0 | 0 | 0 | 0.00% |
|  | − | 20 | 1 | 0 | 0 | 8 | 29 | 61.70% |
|  | 🡫 | 0 | 0 | 0 | 4 | 14 | 18 | 38.30% |
| **Coupling (DCC)** | 🡩 | 0 | 0 | 0 | 0 | 0 | 0 | 0.00% |
|  | − | 20 | 1 | 0 | 4 | 22 | 47 | 100.00% |
|  | 🡫 | 0 | 0 | 0 | 0 | 0 | 0 | 0.00% |
| **Hierarchies (NOH)** | 🡩 | 0 | 0 | 0 | 0 | 0 | 0 | 0.00% |
|  | − | 20 | 1 | 0 | 4 | 22 | 47 | 100.00% |
|  | 🡫 | 0 | 0 | 0 | 0 | 0 | 0 | 0.00% |
| **Abstraction (ANA)** | 🡩 | 0 | 0 | 0 | 0 | 0 | 0 | 0.00% |
|  | − | 20 | 1 | 0 | 4 | 22 | 47 | 100.00% |
|  | 🡫 | 0 | 0 | 0 | 0 | 0 | 0 | 0.00% |
| **Complexity (NOM)** | 🡩 | 0 | 0 | 0 | 4 | 22 | 26 | 55.32% |
|  | − | 20 | 1 | 0 | 0 | 0 | 21 | 44.68% |
|  | 🡫 | 0 | 0 | 0 | 0 | 0 | 0 | 0.00% |

**Table A5: Multi-case analysis of the Rename Method**

| **Internal Quality Attribute** | **Case Study** | **PMS** | **LMS** | **BMS** | **jHotDraw** | **jEdit** | **Total** | **Effect%** |
| --- | --- | --- | --- | --- | --- | --- | --- | --- |
|  | Times | 22 | 5 | 11 | 31 | 40 | 109 |  |
|  | Impact |  |  |  |  |  |  |  |
| **Design Size (DSC)** | 🡩 | 0 | 0 | 0 | 0 | 0 | 0 | 0.00% |
|  | − | 22 | 5 | 11 | 31 | 40 | 109 | 100.00% |
|  | 🡫 | 0 | 0 | 0 | 0 | 0 | 0 | 0.00% |
| **Inheritance (MFA)** | 🡩 | 0 | 0 | 0 | 0 | 0 | 0 | 0.00% |
|  | − | 22 | 5 | 11 | 31 | 40 | 109 | 100.00% |
|  | 🡫 | 0 | 0 | 0 | 0 | 0 | 0 | 0.00% |
| **Messaging (CIS)** | 🡩 | 0 | 0 | 0 | 0 | 0 | 0 | 0.00% |
|  | − | 22 | 5 | 11 | 31 | 40 | 109 | 100.00% |
|  | 🡫 | 0 | 0 | 0 | 0 | 0 | 0 | 0.00% |
| **Composition (MOA)** | 🡩 | 0 | 0 | 0 | 0 | 0 | 0 | 0.00% |
|  | − | 22 | 5 | 11 | 31 | 40 | 109 | 100.00% |
|  | 🡫 | 0 | 0 | 0 | 0 | 0 | 0 | 0.00% |
| **Encapsulation (DAM)** | 🡩 | 0 | 0 | 0 | 0 | 0 | 0 | 0.00% |
|  | − | 22 | 5 | 11 | 31 | 40 | 109 | 100.00% |
|  | 🡫 | 0 | 0 | 0 | 0 | 0 | 0 | 0.00% |
| **Polymorphism (NOP)** | 🡩 | 0 | 0 | 0 | 0 | 0 | 0 | 0.00% |
|  | − | 22 | 5 | 11 | 31 | 40 | 109 | 100.00% |
|  | 🡫 | 0 | 0 | 0 | 0 | 0 | 0 | 0.00% |
| **Cohesion (CAM)** | 🡩 | 0 | 0 | 0 | 0 | 0 | 0 | 0.00% |
|  | − | 22 | 5 | 11 | 31 | 40 | 109 | 100.00% |
|  | 🡫 | 0 | 0 | 0 | 0 | 0 | 0 | 0.00% |
| **Coupling (DCC)** | 🡩 | 0 | 0 | 0 | 0 | 0 | 0 | 0.00% |
|  | − | 22 | 5 | 11 | 31 | 40 | 109 | 100.00% |
|  | 🡫 | 0 | 0 | 0 | 0 | 0 | 0 | 0.00% |
| **Hierarchies (NOH)** | 🡩 | 0 | 0 | 0 | 0 | 0 | 0 | 0.00% |
|  | − | 22 | 5 | 11 | 31 | 40 | 109 | 100.00% |
|  | 🡫 | 0 | 0 | 0 | 0 | 0 | 0 | 0.00% |
| **Abstraction (ANA)** | 🡩 | 0 | 0 | 0 | 0 | 0 | 0 | 0.00% |
|  | − | 22 | 5 | 11 | 31 | 40 | 109 | 100.00% |
|  | 🡫 | 0 | 0 | 0 | 0 | 0 | 0 | 0.00% |
| **Complexity (NOM)** | 🡩 | 0 | 0 | 0 | 0 | 0 | 0 | 0.00% |
|  | − | 22 | 5 | 11 | 31 | 40 | 109 | 100.00% |
|  | 🡫 | 0 | 0 | 0 | 0 | 0 | 0 | 0.00% |

**Table A6: Multi-case analysis of the Introduce Parameter Object**

| **Internal Quality Attribute** | **Case Study** | **PMS** | **LMS** | **BMS** | **jHotDraw** | **jEdit** | **Total** | **Effect%** |
| --- | --- | --- | --- | --- | --- | --- | --- | --- |
|  | Times | 2 | 1 | 2 | 8 | 14 | 27 |  |
|  | Impact |  |  |  |  |  |  |  |
| **Design Size (DSC)** | 🡩 | 2 | 1 | 2 | 8 | 14 | 27 | 100.00% |
|  | − | 0 | 0 | 0 | 0 | 0 | 0 | 0.00% |
|  | 🡫 | 0 | 0 | 0 | 0 | 0 | 0 | 0.00% |
| **Inheritance (MFA)** | 🡩 | 0 | 0 | 0 | 0 | 0 | 0 | 0.00% |
|  | − | 2 | 1 | 2 | 8 | 14 | 27 | 100.00% |
|  | 🡫 | 0 | 0 | 0 | 0 | 0 | 0 | 0.00% |
| **Messaging (CIS)** | 🡩 | 2 | 1 | 2 | 8 | 14 | 27 | 100.00% |
|  | − | 0 | 0 | 0 | 0 | 0 | 0 | 0.00% |
|  | 🡫 | 0 | 0 | 0 | 0 | 0 | 0 | 0.00% |
| **Composition (MOA)** | 🡩 | 0 | 0 | 0 | 0 | 0 | 0 | 0.00% |
|  | − | 2 | 1 | 2 | 8 | 14 | 27 | 100.00% |
|  | 🡫 | 0 | 0 | 0 | 0 | 0 | 0 | 0.00% |
| **Encapsulation (DAM)** | 🡩 | 2 | 1 | 2 | 8 | 14 | 27 | 100.00% |
|  | − | 0 | 0 | 0 | 0 | 0 | 0 | 0.00% |
|  | 🡫 | 0 | 0 | 0 | 0 | 0 | 0 | 0.00% |
| **Polymorphism (NOP)** | 🡩 | 0 | 0 | 0 | 0 | 0 | 0 | 0.00% |
|  | − | 2 | 1 | 2 | 8 | 14 | 27 | 100.00% |
|  | 🡫 | 0 | 0 | 0 | 0 | 0 | 0 | 0.00% |
| **Cohesion (CAM)** | 🡩 | 0 | 0 | 0 | 0 | 0 | 0 | 0.00% |
|  | − | 0 | 0 | 0 | 0 | 0 | 0 | 0.00% |
|  | 🡫 | 2 | 1 | 2 | 8 | 14 | 27 | 100.00% |
| **Coupling (DCC)** | 🡩 | 2 | 1 | 2 | 8 | 14 | 27 | 100.00% |
|  | − | 0 | 0 | 0 | 0 | 0 | 0 | 0.00% |
|  | 🡫 | 0 | 0 | 0 | 0 | 0 | 0 | 0.00% |
| **Hierarchies (NOH)** | 🡩 | 0 | 0 | 0 | 0 | 0 | 0 | 0.00% |
|  | − | 2 | 1 | 2 | 8 | 14 | 27 | 100.00% |
|  | 🡫 | 0 | 0 | 0 | 0 | 0 | 0 | 0.00% |
| **Abstraction (ANA)** | 🡩 | 0 | 0 | 0 | 0 | 0 | 0 | 0.00% |
|  | − | 0 | 0 | 0 | 0 | 0 | 0 | 0.00% |
|  | 🡫 | 2 | 1 | 2 | 8 | 14 | 27 | 100.00% |
| **Complexity (NOM)** | 🡩 | 2 | 1 | 2 | 8 | 14 | 27 | 100.00% |
|  | − | 0 | 0 | 0 | 0 | 0 | 0 | 0.00% |
|  | 🡫 | 0 | 0 | 0 | 0 | 0 | 0 | 0.00% |

**Table A7: Multi-case analysis of the Remove Setting Method**

| **Internal Quality Attribute** | **Case Study** | **PMS** | **LMS** | **BMS** | **jHotDraw** | **jEdit** | **Total** | **Effect%** |
| --- | --- | --- | --- | --- | --- | --- | --- | --- |
|  | Times | 5 | 0 | 26 | 35 | 26 | 92 |  |
|  | Impact |  |  |  |  |  |  |  |
| **Design Size (DSC)** | 🡩 | 0 | 0 | 0 | 0 | 0 | 0 | 0.00% |
|  | − | 5 | 0 | 26 | 35 | 26 | 92 | 100.00% |
|  | 🡫 | 0 | 0 | 0 | 0 | 0 | 0 | 0.00% |
| **Inheritance (MFA)** | 🡩 | 0 | 0 | 0 | 0 | 0 | 0 | 0.00% |
|  | − | 5 | 0 | 26 | 35 | 26 | 92 | 100.00% |
|  | 🡫 | 0 | 0 | 0 | 0 | 0 | 0 | 0.00% |
| **Messaging (CIS)** | 🡩 | 0 | 0 | 0 | 0 | 0 | 0 | 0.00% |
|  | − | 2 | 0 | 16 | 25 | 15 | 58 | 60.87% |
|  | 🡫 | 3 | 0 | 10 | 10 | 11 | 34 | 39.13% |
| **Composition (MOA)** | 🡩 | 0 | 0 | 0 | 0 | 0 | 0 | 0.00% |
|  | − | 5 | 0 | 26 | 35 | 26 | 92 | 100% |
|  | 🡫 | 0 | 0 | 0 | 0 | 0 | 0 | 0.00% |
| **Encapsulation (DAM)** | 🡩 | 0 | 0 | 0 | 0 | 0 | 0 | 0.00% |
|  | − | 5 | 0 | 26 | 35 | 26 | 100 | 100.00% |
|  | 🡫 | 0 | 0 | 0 | 0 | 0 | 0 | 0.00% |
| **Polymorphism (NOP)** | 🡩 | 0 | 0 | 0 | 0 | 0 | 0 | 0.00% |
|  | − | 5 | 0 | 26 | 35 | 26 | 92 | 100.00% |
|  | 🡫 | 0 | 0 | 0 | 0 | 0 | 0 | 0.00% |
| **Cohesion (CAM)** | 🡩 | 2 | 0 | 6 | 25 | 19 | 52 | 54.35% |
|  | − | 0 | 0 | 0 | 0 | 0 | 0 | 0.00% |
|  | 🡫 | 3 | 0 | 20 | 10 | 7 | 40 | 45.65% |
| **Coupling (DCC)** | 🡩 | 0 | 0 | 0 | 0 | 0 | 0 | 0.00% |
|  | − | 5 | 0 | 26 | 35 | 26 | 92 | 100% |
|  | 🡫 | 0 | 0 | 0 | 0 | 0 | 0 | 0.00% |
| **Hierarchies (NOH)** | 🡩 | 0 | 0 | 0 | 0 | 0 | 0 | 0.00% |
|  | − | 5 | 0 | 26 | 35 | 26 | 92 | 100% |
|  | 🡫 | 0 | 0 | 0 | 0 | 0 | 0 | 0.00% |
| **Abstraction (ANA)** | 🡩 | 0 | 0 | 0 | 0 | 0 | 0 | 0.00% |
|  | − | 5 | 0 | 26 | 35 | 26 | 92 | 100% |
|  | 🡫 | 0 | 0 | 0 | 0 | 0 | 0 | 0.00% |
| **Complexity (NOM)** | 🡩 | 0 | 0 | 0 | 0 | 0 | 0 | 0.00% |
|  | − | 0 | 0 | 0 | 0 | 0 | 0 | 0.00% |
|  | 🡫 | 5 | 0 | 26 | 35 | 26 | 92 | 100% |

**Table A8: Multi-case analysis of the Extract Subclass**

| **Internal Quality Attribute** | **Case Study** | **PMS** | **LMS** | **BMS** | **JHotDraw** | **JEdit** | **Total** | **Effect%** |
| --- | --- | --- | --- | --- | --- | --- | --- | --- |
|  | Times | 1 | 2 | 0 | 8 | 29 | 40 |  |
|  | Impact |  |  |  |  |  |  |  |
| **Design Size (DSC)** | 🡩 | 1 | 2 | 0 | 8 | 29 | 40 | 100.00% |
|  | − | 0 | 0 | 0 | 0 | 0 | 0 | 0.00% |
|  | 🡫 | 0 | 0 | 0 | 0 | 0 | 0 | 0.00% |
| **Inheritance (MFA)** | 🡩 | 0 | 0 | 0 | 0 | 0 | 0 | 0.00% |
|  | − | 1 | 2 | 0 | 8 | 29 | 40 | 100.00% |
|  | 🡫 | 0 | 0 | 0 | 0 | 0 | 0 | 0.00% |
| **Messaging (CIS)** | 🡩 | 1 | 0 | 0 | 0 | 27 | 28 | 70.00% |
|  | − | 0 | 2 | 0 | 8 | 2 | 12 | 30.00% |
|  | 🡫 | 0 | 0 | 0 | 0 | 0 | 0 | 0.00% |
| **Composition (MOA)** | 🡩 | 0 | 0 | 0 | 0 | 27 | 27 | 67.50% |
|  | − | 1 | 2 | 0 | 8 | 2 | 13 | 32.50% |
|  | 🡫 | 0 | 0 | 0 | 0 | 0 | 0 | 0.00% |
| **Encapsulation (DAM)** | 🡩 | 1 | 2 | 0 | 8 | 29 | 40 | 100.00% |
|  | − | 0 | 0 | 0 | 0 | 0 | 0 | 0.00% |
|  | 🡫 | 0 | 0 | 0 | 0 | 0 | 0 | 0.00% |
| **Polymorphism (NOP)** | 🡩 | 0 | 0 | 0 | 6 | 16 | 22 | 55.00% |
|  | − | 1 | 2 | 0 | 2 | 13 | 18 | 45.00% |
|  | 🡫 | 0 | 0 | 0 | 0 | 0 | 0 | 0.00% |
| **Cohesion (CAM)** | 🡩 | 1 | 2 | 0 | 8 | 29 | 40 | 100.00% |
|  | − | 0 | 0 | 0 | 0 | 0 | 0 | 0.00% |
|  | 🡫 | 0 | 0 | 0 | 0 | 0 | 0 | 0.00% |
| **Coupling (DCC)** | 🡩 | 0 | 0 | 0 | 2 | 27 | 29 | 72.50% |
|  | − | 1 | 2 | 0 | 6 | 2 | 11 | 27.50% |
|  | 🡫 | 0 | 0 | 0 | 0 | 0 | 0 | 0.00% |
| **Hierarchies (NOH)** | 🡩 | 0 | 2 | 0 | 4 | 24 | 30 | 75.00% |
|  | − | 1 | 0 | 0 | 4 | 5 | 10 | 25.00% |
|  | 🡫 | 0 | 0 | 0 | 0 | 0 | 0 | 0.00% |
| **Abstraction (ANA)** | 🡩 | 1 | 2 | 0 | 8 | 29 | 40 | 100.00% |
|  | − | 0 | 0 | 0 | 0 | 0 | 0 | 0.00% |
|  | 🡫 | 0 | 0 | 0 | 0 | 0 | 0 | 0.00% |
| **Complexity (NOM)** | 🡩 | 1 | 0 | 0 | 6 | 26 | 33 | 82.50% |
|  | − | 0 | 2 | 0 | 2 | 3 | 7 | 17.50% |
|  | 🡫 | 0 | 0 | 0 | 0 | 0 | 0 | 0.00% |

**Table A9: Multi-case analysis of the Extract Superclass**

| **Internal Quality Attribute** | **Case Study** | **PMS** | **LMS** | **BMS** | **jHotDraw** | **jEdit** | **Total** | **Effect%** |
| --- | --- | --- | --- | --- | --- | --- | --- | --- |
|  | Times | 0 | 4 | 4 | 3 | 10 | 21 |  |
|  | Impact |  |  |  |  |  |  |  |
| **Design Size (DSC)** | 🡩 | 0 | 4 | 4 | 3 | 10 | 21 | 100.00% |
|  | − | 0 | 0 | 0 | 0 | 0 | 0 | 0.00% |
|  | 🡫 | 0 | 0 | 0 | 0 | 0 | 0 | 0.00% |
| **Inheritance (MFA)** | 🡩 | 0 | 0 | 0 | 0 | 0 | 0 | 0.00% |
|  | − | 0 | 4 | 4 | 3 | 10 | 21 | 100.00% |
|  | 🡫 | 0 | 0 | 0 | 0 | 0 | 0 | 0.00% |
| **Messaging (CIS)** | 🡩 | 0 | 0 | 0 | 0 | 0 | 0 | 0.00% |
|  | − | 0 | 3 | 1 | 2 | 5 | 11 | 52.38% |
|  | 🡫 | 0 | 1 | 3 | 1 | 5 | 10 | 47.62% |
| **Composition (MOA)** | 🡩 | 0 | 0 | 0 | 0 | 0 | 0 | 0.00% |
|  | − | 0 | 4 | 4 | 3 | 10 | 21 | 100.00% |
|  | 🡫 | 0 | 0 | 0 | 0 | 0 | 0 | 0.00% |
| **Encapsulation (DAM)** | 🡩 | 0 | 4 | 4 | 3 | 10 | 21 | 100.00% |
|  | − | 0 | 0 | 0 | 0 | 0 | 0 | 0.00% |
|  | 🡫 | 0 | 0 | 0 | 0 | 0 | 0 | 0.00% |
| **Polymorphism (NOP)** | 🡩 | 0 | 0 | 0 | 0 | 0 | 0 | 0.00% |
|  | − | 0 | 4 | 4 | 3 | 10 | 21 | 100.00% |
|  | 🡫 | 0 | 0 | 0 | 0 | 0 | 0 | 0.00% |
| **Cohesion (CAM)** | 🡩 | 0 | 0 | 3 | 3 | 6 | 12 | 57.14% |
|  | − | 0 | 4 | 1 | 0 | 4 | 9 | 42.86% |
|  | 🡫 | 0 | 0 | 0 | 0 | 0 | 0 | 0.00% |
| **Coupling (DCC)** | 🡩 | 0 | 0 | 0 | 0 | 0 | 0 | 0.00% |
|  | − | 0 | 4 | 4 | 3 | 10 | 21 | 100.00% |
|  | 🡫 | 0 | 0 | 0 | 0 | 0 | 0 | 0.00% |
| **Hierarchies (NOH)** | 🡩 | 0 | 4 | 4 | 3 | 10 | 21 | 100.00% |
|  | − | 0 | 0 | 0 | 0 | 0 | 0 | 0.00% |
|  | 🡫 | 0 | 0 | 0 | 0 | 0 | 0 | 0.00% |
| **Abstraction (ANA)** | 🡩 | 0 | 4 | 4 | 3 | 10 | 21 | 100.00% |
|  | − | 0 | 0 | 0 | 0 | 0 | 0 | 0.00% |
|  | 🡫 | 0 | 0 | 0 | 0 | 0 | 0 | 0.00% |
| **Complexity (NOM)** | 🡩 | 0 | 0 | 0 | 0 | 0 | 0 | 0.00% |
|  | − | 0 | 0 | 0 | 0 | 0 | 0 | 0.00% |
|  | 🡫 | 0 | 4 | 4 | 3 | 10 | 21 | 100.00% |

**Table A10: Multi-case analysis of the Extract Interface**

| **Internal Quality Attribute** | **Case Study** | **PMS** | **LMS** | **BMS** | **jHotDraw** | **jEdit** | **Total** | **Effect%** |
| --- | --- | --- | --- | --- | --- | --- | --- | --- |
|  | Times | 2 | 0 | 4 | 10 | 10 | 26 |  |
|  | Impact |  |  |  |  |  |  |  |
| **Design Size (DSC)** | 🡩 | 2 | 0 | 4 | 10 | 10 | 26 | 100.00% |
|  | − | 0 | 0 | 0 | 0 | 0 | 0 | 0.00% |
|  | 🡫 | 0 | 0 | 0 | 0 | 0 | 0 | 0.00% |
| **Inheritance (MFA)** | 🡩 | 0 | 0 | 0 | 0 | 0 | 0 | 0.00% |
|  | − | 2 | 0 | 4 | 10 | 10 | 26 | 100.00% |
|  | 🡫 | 0 | 0 | 0 | 0 | 0 | 0 | 0.00% |
| **Messaging (CIS)** | 🡩 | 2 | 0 | 4 | 10 | 10 | 26 | 100.00% |
|  | − | 0 | 0 | 0 | 0 | 0 | 0 | 0.00% |
|  | 🡫 | 0 | 0 | 0 | 0 | 0 | 0 | 0.00% |
| **Composition (MOA)** | 🡩 | 0 | 0 | 0 | 0 | 0 | 0 | 0.00% |
|  | − | 2 | 0 | 4 | 10 | 10 | 26 | 100.00% |
|  | 🡫 | 0 | 0 | 0 | 0 | 0 | 0 | 0.00% |
| **Encapsulation (DAM)** | 🡩 | 0 | 0 | 0 | 0 | 0 | 0 | 0.00% |
|  | − | 2 | 0 | 4 | 10 | 10 | 26 | 100.00% |
|  | 🡫 | 0 | 0 | 0 | 0 | 0 | 0 | 0.00% |
| **Polymorphism (NOP)** | 🡩 | 0 | 0 | 0 | 0 | 0 | 0 | 0.00% |
|  | − | 2 | 0 | 4 | 10 | 10 | 26 | 100.00% |
|  | 🡫 | 0 | 0 | 0 | 0 | 0 | 0 | 0.00% |
| **Cohesion (CAM)** | 🡩 | 2 | 0 | 4 | 10 | 10 | 26 | 100.00% |
|  | − | 0 | 0 | 0 | 0 | 0 | 0 | 0.00% |
|  | 🡫 | 0 | 0 | 0 | 0 | 0 | 0 | 0.00% |
| **Coupling (DCC)** | 🡩 | 0 | 0 | 0 | 5 | 1 | 6 | 23.08% |
|  | − | 2 | 0 | 4 | 5 | 9 | 20 | 76.92% |
|  | 🡫 | 0 | 0 | 0 | 0 | 0 | 0 | 0.00% |
| **Hierarchies (NOH)** | 🡩 | 2 | 0 | 4 | 10 | 10 | 26 | 100.00% |
|  | − | 0 | 0 | 0 | 0 | 0 | 0 | 0.00% |
|  | 🡫 | 0 | 0 | 0 | 0 | 0 | 0 | 0.00% |
| **Abstraction (ANA)** | 🡩 | 0 | 0 | 0 | 0 | 0 | 0 | 0.00% |
|  | − | 0 | 0 | 0 | 0 | 0 | 0 | 0.00% |
|  | 🡫 | 2 | 0 | 4 | 10 | 10 | 26 | 100.00% |
| **Complexity (NOM)** | 🡩 | 2 | 0 | 4 | 10 | 10 | 26 | 100.00% |
|  | − | 0 | 0 | 0 | 0 | 0 | 0 | 0.00% |
|  | 🡫 | 0 | 0 | 0 | 0 | 0 | 0 | 0.00% |
